# Childhood tuberculosis: management and treatment outcomes among children in Northwest Ethiopia: a cross-sectional study

**DOI:** 10.11604/pamj.2017.27.25.10120

**Published:** 2017-05-10

**Authors:** Zemene Tigabu Kebede, Belaynew Wasie Taye, Yohannes Hailu Matebe

**Affiliations:** 1 Department of Paediatrics, College of Medicine, University of Gondar, Ethiopia; 2 School of Public Health, College of Medicine, Bahir Dar University, Ethiopia

**Keywords:** Treatment outcomes, determinants, children, developing country

## Abstract

**Introduction:**

Childhood tuberculosis (TB) treatment is becoming a major challenge in the TB control efforts of the Ethiopian health system. This study assessed childhood tuberculosis management, and treatment outcomes among children who completed anti-TB treatment in Northwest Ethiopia.

**Methods:**

A cross-sectional study was conducted among children who completed their anti-TB treatment in Gondar University Referral Hospital and 6 satellite health centers. Data from each child with tuberculosis were obtained from review of medical records. P-values < 0.05 were considered statistically significant.

**Results:**

The commonest method of childhood TB diagnosis was clinical assessment combined with chest x-ray (48.5%). Absence of compliance with TB treatment guideline (98.7%), providing inadequate anti-TB regimen (1.8%), and poor adherence to treatment (22.5%) were challenges in management of childhood tuberculosis. Treatment success rate was 78.9%. In the bivariate regression, factors associated with TB treatment outcomes were permanent residence (OR=8.3, 95%CI: 4.1, 16.7), antiretroviral therapy (OR=4.5, 95%CI: 1.2, 16), and adherence to treatment (p < 0.001). After controlling for confounders, adherence to anti-TB treatment (OR=0.003, 95% CI: 0.001, 0.02) was independent predictor of treatment success.

**Conclusion:**

Anti-tuberculosis treatment success rate was still low among children in Northwest Ethiopia. The health centers and hospital shall enhance strong follow-up of children on anti-tuberculosis treatment to improve treatment success with focus on rural children.

## Introduction

Ethiopia is one of the high tuberculosis (TB)/human immunodeficiency virus (HIV) and multidrugs resistant (MDR) TB burden countries. According to the 2014 World Health Organization (WHO) report, the prevalence and incidence of all forms of TB were 211 and 224 per 100,000 populations respectively. According to the recent national TB drug resistance surveillance report, 2.3% of new TB cases and 17.8% of previously treated TB cases were estimated to have MDR TB [1]. The extent of childhood TB is unknown but estimated between 9.6% and 11% out of all incident cases, with the majority of cases occurring in high TB burden countries. The difficulties in establishing a definitive diagnosis, the presence of extra-pulmonary disease, and the lower priority paid to smear negative TB are among many challenges to estimate the burden of TB in children [2]. The national TB prevalence is 572 cases per 1000 population and its incidence is 359 new cases per 1000 population at risk. Ethiopia has a low case detection rate of about 50% (42%–62%) [3]. Health institution reports revealed childhood TB constitutes up to 47% of all TB cases. Among children in Ethiopia, smear negative TB is the dominant type with a frequency of 56-65% followed by extra-pulmonary TB (24-45%) and smear positive TB with prevalence of all forms of TB about 20% [4, 5]. TB in children is affected by weight loss which is commonly due to under-nutrition and HIV infection. There is a need for more aggressive evaluation for TB among these groups of children [6, 7]. With an increasing trend of MDR TB in the general population including children, assessing the risks for MDR TB is vital for the health care settings. There are studies on incidence and treatment outcome of TB in HIV infected patients and adults [8], but little evidence is available on the treatment outcomes and potential risks for MDR TB among children. This study, therefore, assessed the management, anti-TB treatment outcomes, and factors associated with treatment outcomes among children who completed their anti-TB treatment in the last 1 year at a referral hospital and 6 health centers.

## Methods

**Study design:** a cross-sectional study was conducted among children who completed anti-TB treatment in a referral hospital and six health centers in Northwest Ethiopia from September 2014 – March 2015.

**Study setting:** the study was conducted in Gondar University Referral Hospital (GURH) and 6 satellite health centers. The GURH is a tertiary care and teaching hospital that has more than 478 beds of which 75 belong to the paediatric ward. TB is the third common cause of admission in the hospital. The hospital has a separate TB clinic that diagnoses, initiates treatment and follows-up for the period of treatment to see outcomes. Annually, about 120 children are treated for TB. The health centers are the central facilities in the primary care unit. The health centers also diagnose and treat children for TB. In addition, they serve children referred from hospitals for the purpose of follow-up as they are closer to the households than the referral hospital. Physicians, health officers (bachelor medical officers), and nurses perform diagnosis and treatment of TB at health centers and hospital [9, 10].

**Study population:** the initial sample was determined using formula for estimation of single population proportions with z statistic=1.96, margin of error, w=0.05, proportion of treatment success, p= 85.5% [11] and non-response rate of 10%. Since sample size is nearly similar to total number of children who took anti TB, all 227 children below age 15 years who completed anti-TB treatment in the last one year at GURH and the 6 satellite health centers were studied. However, children started anti-TB at the hospital and referred to other health facility for further follow-up and referral cases to the study facilities for special work-up consultations were excluded as these children will not complete their follow-up in the study sites to see the outcome of treatment.

**Study variables:** the dependent variable is treatment outcome. Associated variables of the study included socio-demographic characters (age, sex, family residence, presence of parents),co-morbidity (stage of HIV disease, duration of HIV infection, history of TB in family, history of MDR TB in family, age at diagnosis of HIV infection),and treatment related factors (anti-retroviral treatment (ART) status, anti-TB treatment regimen, adherence, duration of therapy).

**Operational definitions:** treatment success was defined as completion of full course of anti-TB with absence of symptoms and signs of TB or a child after completion are declared cured. Adherence was defined the extent to which a child on anti-TB is compliant to treatment. *Good adherence* was defined as adherence rate of 85% or above while those below were defined as having *poor adherence to treatment* [12].

### Data collection procedures

A pre-tested data extracted form developed in English, which contains socio-demographic characteristics, family conditions, HIV status, anti TB adherence, and co-morbidities was used to collect data on children and parents. Data were collected by clinicians practicing in the respective health facilities as clinical knowledge and onsite deployment were necessary for feasibility reasons. A close supervision was conducted throughout data collection along with cross-checking of filled data to minimize social desirability bias. Prior to actual data collection, training was provided to data collectors on the data collection techniques used and to familiarize the data extraction tool. The data extraction tools were prepared from the review of similar literature (9…) The data collectors extracted the required information from charts of children whose treatment status was known. The investigators supervised data collection directly during each day of data collection and checked the filled forms for completeness and accuracy. A code was used for children and guardians during data collection to maintain confidentiality of information.

### Statistical analysis

Data were entered in to Epi Info version 3.5.3 software for windows and analysed using statistical package for social sciences (SPSS) version 20.0 statistical package. Descriptive statistics were used to describe basic characteristics of children and guardians and comorbidities of children. The proportion of outcomes of anti-TB treatment was also computed. Bivariate and multivariable logistic regression analyses were calculated to identify independent predictors of treatment success. *P-values* less than 0.05 and 95% CI not including the null value 1 were considered as statistically significant.

### Ethical issues

The study was conducted after ethical approval was obtained from Institutional Review Board of the University of Gondar. Letter of permission to data collection was obtained from the offices of medical director and heads of health centers and delivered to the departments /clinics serving children. Informed consent was obtained from the guardian/caregiver of each child after detailed explanation and confirmation of understanding. To maintain confidentiality of information, the questionnaires were anonymous and code numbers were used to identify each child. Caregivers were given brief education on adherence to treatment and prevention of TB transmission after each interview.

## Results

### Socio-demographic characteristics

**Table 1** presents the basic characteristics of children and caregivers. A total of 227 (54.2% male and 45.8% female) children were studied. The mean (+ s.d) age of children, at start of treatment, was 6.8(+ 4.1) years with the age range of 2 months to 14 years. Nearly three-forth (73.6%) were from urban residence. A quarter (24.7%) of TB infected children were HIV positive (Table 1).

**Table 1:** Basic characteristics of children who completed anti tuberculosis treatment in Northwest Ethiopia, September 2014-March 2015

Characteristic	Number	Percent
**Age group of children(years): (mean=6.8, st.d.=4.1)**		
</=1	25	11.0
2-5	64	28.2
6-10	87	38.3
11-15	51	22.5
**Sex**		
Male	123	54.2
Female	104	45.8
Residence		
Urban	167	73.6
**Rural**	60	26.4
**HIV infection in the child**		
Yes	56	24.6
No	135	59.5
Status not known	36	15.9
**ART status (n=56)**		
On ART	31	55.4
Not on ART	25	44.6

### Tuberculosis diagnosis and treatment among children in Northwest Ethiopia

The commonest diagnostic procedures followed were chest x-ray with clinical assessment comprising of nearly half (48.5%) of children diagnosed for TB. Sputum testing with chest x-ray and clinical assessment were used in 8 (3.5%) children diagnosed with TB. Almost all, 221 (97.4%) children were started on first line regimen. Only one child was initiated with retreatment regimen. Among children who were followed for the course of treatment, 48(21.1%) were defaulters while 170 (74.9%) completed treatment and 9(4.0%) of them were declared cured. The overall treatment success rate was 78.9% (Table 2).

**Table 2:** Tuberculosis diagnosis and treatment related characteristics of children treated for tuberculosis in Northwest Ethiopia, September 2014-March 2015

Characteristic	Number	Percent
**Diagnosis technique**		
CXR plus clinical	110	48.5
FNAC + CXR +clinical	22	9.7
Sputum +CXR +clinical	8	3.5
Contact Hx + CXR + clinical	11	4.8
Sign and Symptoms alone	2	0.9
Others	74	32.6
**Type of treatment**		
New treatment regimen	221	97.4
Retreatment regimen	1	0.4
Others	5	2.2
**Past history of treatment**		
Yes	13	5.7
No	214	94.3
Type of drug currently taken		
RHZE +RH	226	99.6
Intensive phase other than RHZE +RH	1	0.4
**TB treatment outcome**		
Cured	9	4.0
Completed	170	74.9
Defaulter	48	21.1
**TB treatment success**		
Yes	179	78.9
No	48	21.1

### Challenges in management of childhood tuberculosis

**Table 3** presents the challenges in management of childhood tuberculosis in the paediatric TB clinics. The guidelines for treatment of children with TB were at hand in all health facilities but there was no compliance to the national guidelines, in 98.7% of the cases, where the recommendations for diagnosis and treatment were not strictly followed. Training, supervision and treatment monitoring were there in all of the cases. The regimens provided were adequate (full combination of drugs for adequate period of time) in 223(98.2%) of children treated for TB while inadequate regimens were used in 4 (1.8%) children. Other factors that may predispose to MDR TB were poor storage of drugs (0.4%), poor adherence to treatment, (22.5%), and presence of side effects of anti TB drugs (0.9%) (Table 3).

**Table 3:** Health care provider and drug related challenges in TB treatment among children in Northwest Ethiopia, September 2014-March 2015

Character	Number	Percent
**National Tb treatment guideline at hand while treating child**		
Yes	227	100.00
No	0	0
**Health workers complied with the guideline while diagnosing and treating child\**		
Yes	3	1.3
No	224	98.7
Health workers trained on national guideline treated child	227	100.0
Presence of regular supportive supervision	7	100.0
Regular monitoring treatment provision	7	100.0
**Anti-TB Regimen is adequate**		
Yes	223	98.2
No	4	1.8
**All regimens available in paediatric preparations**		
Yes	7	100.0
No	0	0
**Poor storage condition**		
Yes	1	0.4
No	6	99.6
**Adherence to treatment**		
Poor	51	22.5
Good	176	77.5
**Health education given to child /caregiver/**		
Yes	227	100.00
No	0	0
**Lack of transportation/money support**		
Yes	2	0.9
No	225	99.1
**Drug adverse effect observed**		
Yes	2	0.9
No	225	99.1

### Factors associated with TB treatment outcomes

The factors associated with treatment outcomes among children taking anti TB are presented in Table 4. Permanent residence of child and hence family was significantly associated with treatment success in the bivariate analysis (crude odds ratio (COR)=8.3, 95% CI: 4.1, 16.7). In the multivariable regression, urban residents were 1.2 times more likely to be cured/ treatment completed as compared to rural children but was not statistically significant (adjusted odds ratio(AOR)=1.2, 95% CI: 0.24,5.93). Children from urban areas had a treatment success rate of 89.2% while among rural children treatment success rate was 50%.

**Table 4:** Bivariate and multivariable logistic regression analysis of factors associated with anti-Tuberculosis treatment outcomes among children in Northwest Ethiopia, September 2014-March 2015

Independent variables	Treatment outcome		Crude Odds ratio (95% CI)	Adjusted Odds Ratio (95% C.I.)
	Cured /completed	Defaulter		
**Residence of children**				
Urban	149	18	8.3 (4.1, 16.7)	1.2(0.24, 5.93)
Rural	30	30	1	1
**Age group of children**				
<5years	57	20	0.7(0.34,1.26)	0.99(0.26, 3.77)
>5years	122	28	1	1
**Adherence to treatment**				
Good	171	5	1	1
Poor	8	43	0.005 (0.002, 0.017)	0.003(0.001, 0.02)
**HAART status**				
On ART	27	4	4.5(1.2, 16)	0.43 (0.5, 3.4)
Not on ART	15	10	1	1
**HIV status**				
Have HIV infection	42	14	1.68(0.7, 4.1)	2.2 (0.3, 16)
Have no HIV infection	115	20	3.14(1.4, 7.0)	1.4 (0.3, 7.3)
Status not known	22	14	1	1

Children under the age of 5 years were less likely to have treatment completed or cured as compared to children > 5 years. There was no significant association between age group of children and treatment outcomes (AOR=0.99, 95% CI: 0.26, 3.77). Children without HIV infection were more than 3times more likely to complete treatment or cured from TB than those with unknown HIV status (OR=3.14, 95% CI: 1.4, 7.0). Among HIV infected children TB treatment defaulter rate was 25% while among HIV negative children the defaulter rate was 15.2%. Children who have poor adherence to anti TB treatment and care were more than 90% less likely to cure from TB as compared to TB infected children with good treatment adherence (Table 4).

## Discussion

This study assessed the methods of diagnosis used to diagnose TB among children, the challenges of management, treatment outcomes and determinants among TB infected children. The commonest diagnostic method in this study was chest x-ray combined with clinical evaluation (48.5%). This is because pulmonary TB is the commonest TB in children [4, 5]. The other reasons are the difficulty to get sputum samples from children, rarity of smear positive TB in children, and inability to do Acid Fast Staining which is the gold standard in the diagnosis of childhood TB [2]. The study also employed sign and symptoms only as diagnostic method in 2 children. This is because of the urgency in the need for diagnosis of TB before the laboratory tests and x-ray examinations are ready to be used.

The type of anti-TB treatment among children was predominantly new treatment regimen (97%) because children did not have longer exposure and majority didn’t have past treatment history (Table 2). A study in Addis Ababa on a five years retrospective study also found the majority (88.1%) of children were registered as new TB patients whereas twenty-three (0.9%) children with TB were retreatment cases [11]. This similar finding from another Ethiopian setting signifies that the commonest type is new treatment category in Ethiopia.

The anti TB treatment success rate among children in this study was 78.9%. This is lower than a study from Addis Ababa [11] where 85.5% were successfully treated. This discrepancy may be due to the access to distance from the health facilities is favourable in the Addis Ababa study while our study included 60 (26.4%) children from rural areas and children have to pay for transport and travel long to get to the health centers (Table 1).

In this study the proportion of TB treatment success rate among urban children (89.2%) is comparable to the Addis Ababa study. This shows us that success rates among rural children are lower and need special attention. A study from Gondar University Referral Hospital [13] also found a higher proportion (90%) of treatment success rates among children with TB than this study. This may be due to the fact that the GURH study included children only from the tertiary care center with better diagnostic and treatment facilities. This study, however, included children from health centers with only minimal primary health care packages that may lead to poorer success rates and increased defaulter rates.

A study from Sidama zone has revealed a similar treatment success rate (77%) for children with tuberculosis [5]. The similar rates may be due to inclusion of multiple centers with children having similar backgrounds of socioeconomic status, distance from health facility. The anti TB treatment defaulter rate among children in this study was 21.1%. This is a very high rate as compared to studies elsewhere. Studies from Addis Ababa (3.8%) [11], Southern Ethiopia [5] identified lower defaulter rates than this study. This is because the study participants from the Addis Ababa study were from urban area where they have closer access to collect drugs and adhere to appointment for care.

A systematic review of compliance to anti-TB treatment and risk factors found out the proportion of patients defaulting varied from 11.3% (8) to 29.6% from different studies [14]. This is in line with our study finding of 21.1%. This reflects the contexts of studies are similar the being in Sub Saharan Africa and inclusion of populations that are related to the study participants of our study.

Residence of the child and caregiver was significantly associated with TB treatment outcomes of the child. Children who were from rural areas were 8.3 times more likely to default from treatment than children from the urban areas (crude odds ratio (COR) =8.3, 95%CI: 4.1, 16.7). The major reason to more defaulter rates in rural children is the distance from the health facility is far and may not reach in time to collect the INH drugs. The other reason is that the rural children and care takers may not have money to pay for the round trip transportation and could miss schedules. A review of TB treatment compliance in Sub Saharan Africa also identified distance from health facility as an important determinant of treatment success [14].

The study also found out that persons who used public transport were more likely to be defaulters of anti TB treatment as compared to persons using private or rented transportation systems. This signifies the role of physical accessibility to treatment in improving outcomes of therapy. This tells us the importance of task shifting to initiate IPT at lower level facilities to reach persons from rural areas.

HIV infection increased the risk of defaulter from anti TB treatment. HIV infection causes additional morbidity and discomfort leading to lesser adherence to treatment. Hailu, et’al also found out that HIV infection and unknown HIV status were predictors of poor treatment outcomes [11]. This may be due to the opportunity to treat children with HIV infection and higher chance of disclosure of their HIV status that leads to better adherence to treatment. Study from Nigeria also revealed an increased mortality from HIV infected TB patients [15]. Another study from Kenya also found HIV co-infection is an independent predictor of default from treatment. HIV infected persons were 1.56 times more likely to be defaulters from treatment than non-infected ones [16].

Antiretroviral therapy improves treatment completion/cure from TB (COR=4.5, 95%CI: 1.2, 16) in the bivariate analysis, but turned out to be non-significant and even negatively associated in multivariable regression. This was due to the effect of confounding variables. The negative association reflects the possible pill burden and adverse effects of both anti-TB and ART drugs among children who are taking anti-retroviral medication. Due to the nature of data collection more independent variables couldn’t be explored through different data collection methods and sources and were not tested.

## Conclusion

The commonest methods of diagnosis of childhood TB were clinical assessment with chest x-ray which lacks in almost all health centers in Ethiopia due to absence of x-ray facility. TB treatment success rate is still low in Northwest Ethiopia. Lack of follow up of national guideline, lack of anti TB regimen and poor adherence to treatment were major challenges in the management of TB among children. Adherence to anti-TB treatment was an independent predictor of treatment success. Health centers and district health offices shall work hard to improve compliance to national TB treatment guideline. Children started on anti-TB shall be linked to the nearby health facility to improve treatment success. Focus shall be given to rural children and those with HIV infection.

### What is known about this topic

- Success of childhood TB treatment is determined by undernutrition and HIV infection;
- Emergence of MDR TB causes additional challenge to childhood TB treatment.

### What this study adds

- Low level of adherence to the national TB treatment guideline by health care workers in treating child TB;
- Antiretroviral therapy is associated with the treatment completion/cure from TB;
- Adherence to anti-TB treatment is independent predictor of treatment success.

## Competing interests

The authors declare no competing interest.
